# Nonlinear increase of X-ray intensities from thin foils irradiated with a 200 TW femtosecond laser

**DOI:** 10.1038/srep13436

**Published:** 2015-09-02

**Authors:** A. Ya. Faenov, J. Colgan, S. B. Hansen, A. Zhidkov, T. A. Pikuz, M. Nishiuchi, S. A. Pikuz, I. Yu. Skobelev, J. Abdallah, H. Sakaki, A. Sagisaka, A. S. Pirozhkov, K. Ogura, Y. Fukuda, M. Kanasaki, N. Hasegawa, M. Nishikino, M. Kando, Y. Watanabe, T. Kawachi, S. Masuda, T. Hosokai, R. Kodama, K. Kondo

**Affiliations:** 1 Institute for Academic Initiatives, Osaka University, Suita, Osaka, 565-0871, Japan; 2 Joint Institute for High Temperatures, Russian Academy of Sciences, Moscow 125412, Russia; 3 Theoretical Division, Los Alamos National Laboratory, Los Alamos, NM 87545, USA; 4 Sandia National Laboratories, Albuquerque, New Mexico 87123, USA; 5 PPC and Graduate School of Engineering, Osaka University, 2-1, Yamadaoka, Suita, Osaka 565-0871, Japan; 6 Quantum Beam Science Directorate, Japan Atomic Energy Agency, Kizugawa, Kyoto, Japan; 7 National Research Nuclear University (MEPhI), Moscow 115409, Russia; 8 Interdisciplinary Graduate School of Engineering Sciences, Kyushu University, Japan

## Abstract

We report, for the first time, that the energy of femtosecond optical laser pulses, *E*, with relativistic intensities *I* > 10^21^  W/cm^2^ is efficiently converted to X-ray radiation, which is emitted by “hot” electron component in collision-less processes and heats the solid density plasma periphery. As shown by direct high-resolution spectroscopic measurements X-ray radiation from plasma periphery exhibits unusual non-linear growth ~*E*^*4–5*^ of its power. The non-linear power growth occurs far earlier than the known regime when the radiation reaction dominates particle motion (RDR). Nevertheless, the radiation is shown to dominate the kinetics of the plasma periphery, changing in this regime (now labeled RDKR) the physical picture of the laser plasma interaction. Although in the experiments reported here we demonstrated by observation of KK hollow ions that X-ray intensities in the keV range exceeds ~10^17^  W/cm^2^, there is no theoretical limit of the radiation power. Therefore, such powerful X-ray sources can produce and probe exotic material states with high densities and multiple inner-shell electron excitations even for higher *Z* elements. Femtosecond laser-produced plasmas may thus provide unique ultra-bright X-ray sources, for future studies of matter in extreme conditions, material science studies, and radiography of biological systems.

There are number of platforms for the creation of extremely bright and short X-ray beams^1^ necessary for biological imaging^2^, for material processing^3^,^4^,^5^,^6^, for general physics such as laboratory astrophysics^7^,^8^,^9^,^10^,^11^,^12^, studies of high energy density plasma^13^,^14^ and exotic states of matter^15^,^16^,^17^. The list of powerful X-ray sources has increased in recent years to include transient-collisional plasma X-ray lasers^18^,^19^, powerful X-ray free-electron-laser sources (XFELs)^15^,^20^,^21^, high order harmonics from plasma^22^,^23^,^24^,^25^, Compton scattering extending X-ray energies into sub-MeV range^26^,^27^,^28^, and coherent synchrotron radiation including table-top laser systems^29^,^30^,^31^. Recently it was proposed that radiation from the dense plasma generated by Petawatt-class laser femtosecond pulses incident on thin-foil targets could be used to explore the radiation dominant kinetic regime (RDKR)^17^.

The RDKR is that regime where the radiation intensity dominates standard collisional atomic processes, thereby creating exotic states of matter composed of hollow atoms with multiple inner-shell excitations, which are diagnosed through the observation of unique spectral lines^15^,^17^,^32^,^33^. (Note, that plasma produced by XFEL radiation^15^ is a typical sample of RDKR). The RDKR occurs at far lower laser intensities than the radiation damping regime (RDR) where radiation dominates particle dynamics^34^,^35^,^36^,^37^. Although Petawatt-class lasers (PWL) with a flux density of 10^21^ W/cm^2^ or more are not yet commercial, they already exist in multiple laboratories around the world.

The basic idea of RDKR by PWL irradiation is the following. Ultra-relativistic laser light of high power is focused onto a thin metal foil, creating a central Zone at the laser focus where energetic electrons and ions can interact to produce bright radiation (see below). Surrounding peripheral Zones are subjected to the X-ray flux from the central Zone, where effects of energetic particles are less important^38^,^39^,^40^,^41^. In the central Zone, collisional radiation such as Bremsstrahlung and photo-recombination radiation is typically weak and increases linearly with the laser pulse energy. However, due to the optical field ionization, valence electrons are ionized and then are quickly accelerated to high (MeV) energies. These electrons drive ion acceleration, resulting in rapid plasma expansion, and radiate X-ray photons up to very high energies during their multiple passing through the foil (the effect is known as refluxing). The power of plasma radiation in this case can be characterized by a parameter: 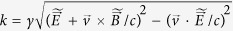, which resembles the quantum radiation parameter *χ*^42^. Here *γ* and *v* are an average relativistic factor and velocity for plasma electrons; *E* and *B* are the total electric and magnetic fields strength. The larger the parameter *k*, the stronger the plasma radiation. Possible sources of X-ray emission include: (i) Compton scattering in a laser pulse: 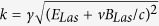, (ii) cyclotron radiation in an azimuthal magnetic field located near plasma edges: 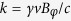, and (iii) harmonic radiation in a transverse plasma electric field occurring in a curved plasma: 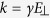. Typically, the strengths of plasma magnetic and electric fields are proportional to the laser field strength: *a*_0_ = *eE*_las_/*mcω* (*ω* is the laser frequency) while an average relativistic factor^43^,^44^ 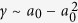. Since the radiation power is proportional to *k*^2^, it scales as 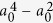 for a single electron. The radiation power thus rapidly approaches the total laser pulse power as *a*_0_ increases. The number of radiating electrons also increases with the laser intensity weakly depending on target conditions^45^. For PWL intensities above 10^22^ W/cm^2^ the radiation process begins to dominate over the electron dynamics, reaching the RDR plasma Zone, where the interaction occurs. However, the RDKR may play an important role even at much lower laser intensities. X-rays from the central, hot, collision-less plasma Zone (See Fig. 1) irradiate the peripheral plasma (Zone 1–3) resulting in its heating and pumping of inner-shell transitions in atoms and ions. If such heating and pumping becomes dominant in the plasma kinetics, the RDKR is achieved. It is of high interest and importance to understand experimentally and theoretically at what intensities radiation effects become dominant.

The X-ray source produced in the relativistic laser interaction with plasma approaching the RDKR has high temporal and spatial non-uniformity. The duration of the radiation from central collision-less plasma Zone (Fig. 1), produced by the interaction of energetic electrons with plasma fields, is approximately the scale of the laser pulse duration or slightly longer, i.e. ~100 fs. The secondary X-ray radiation from periphery plasma, pumped and heated by the X-rays from the central part, has a much longer duration. Moreover, the spectral characteristics of the radiation from the central part and from the periphery, which are determined by different processes, are recognizably distinct. Due to the time- and spatial-integrated technique typically used for X-ray spectra measurements and the rather narrow angle of observation, all of the above mentioned processes contribute to the total measured X-ray power and the influence of various processes involved is difficult to separate experimentally. Nevertheless, measurements of the X-ray spectra with very high spectral resolution (λ/δλ *~* 3000) in a rather wide spectral range, under laser irradiation of foils with different intensities, furnish insights into the nature of radiation processes for both the central and peripheral parts of the targets. Coupled with sophisticated non-LTE kinetics calculations using the collisional-radiative codes ATOMIC^17^,^46^ and SCRAM^32^,^47^,^48^, which include all relevant atomic processes, (photoionization, collisional ionization, auto-ionization, collisional and radiative excitation and de-excitation, and all recombination processes), such measurements allow for spectral separation of radiation emitted from central and periphery plasma.

In most previous experiments with moderate laser intensities below 10^20^ W/cm^2^ the effects of radiation appeared to be negligibly small. Evidence for extremely high power of X-ray radiation and RDKR formation for laser intensities exceeding 10^20^ W/cm^2^ was reported in Ref. 17, but up to now, no systematic measurements of X-ray power behavior vs laser intensity approaching and forming the RDKR has been provided, particularly for ultra-short femtosecond laser pulse duration. While shortening the pulse duration allows higher laser intensities and higher temperature and density of energetic electrons, the dynamics and efficiency of X-rays may differ from the long pulse case presented in Ref. 17. Here, via high resolution X-ray spectroscopy measurements and atomic calculations, we, for the first time, demonstrate that X-ray intensity growth from solid periphery of femtosecond laser plasmas is extremely non-linear, scaling with the laser pulse energy *E* as ~ *E*^4–5^. This non-linearity can be explained only by non-linear processes: an increase of X-ray radiation power from the central part of collision-less plasma with increasing laser intensity. For sub-PWL intensities approaching ~10^21^ W/cm^2^ the pumping X-ray intensity must exceed 10^17^ W/cm^2^ to heat electrons in the solid periphery up to temperatures about 300 eV. Such temperatures are necessary to fit the theoretical and experimental spectra of the X-ray continuum. The same value of intensity of pumping X-rays is necessary to fit spectra of hollow ions for that laser power. Moreover, the best fitting of calculated and measurement results requires the spectral characteristics of pumping X-ray source close to two parametric Macdonald functions typical for X-ray generation in hot, collision-less plasma.

## Results

### Measurements of X-ray and electron spectra

The measurements were made at the J-KAREN laser facility of the Kansai Photon Science Institute of the Japan Atomic Energy Agency^49^. Spectrally resolved X-ray emission in the energy ranges 1450–1850 eV and 2210–2730 eV were investigated (see a scheme of experiment in Fig. 2(a) from aluminium foils of 0.8, 2, 3 and 6 μm thicknesses irradiated by 35 fs Ti:Saph laser pulses with energies from 0.4 J to 7 J. All experimental data have been obtained in a single laser shot (see for more details Methods section) Typical Al K-shell spectra are shown in Fig. 2(b). The green, yellow, black, and red curves represent the data obtained from 6, 3, 2 and 0.8 μm Al foils, respectively, irradiated by laser pulses with 6.8–7.0 J on the target—the maximum available. In such case the laser intensity at the central area of the focal spot of 4.5 μm diameter reached ~1.0 × 10^21^ W/cm^2^. For comparison, spectra observed for lower pulse energies, down to 0.46 J, from Al target with thickness of 0.8 μm are also presented. We should stress that due to a very high intensity of plasma radiation presented in Fig. 2(b) spectra were measured in a single laser shot despite the fact that X-ray spectrometer detector was placed at the distance > 2000 mm from the plasma. A strong, non-linear dependence of the X-ray radiation power on the energy of incident laser beams is clearly observed in Figs. 2(b) and 3.

The total X-ray intensity was measured by an X-ray CCD as a sum of intensities in the energy ranges ~1450–1850 eV and 2210–2730 eV. Usually, the X-ray intensity in this range grows very slowly and almost linearly with the laser pulse intensities (or pulse energies)^38^,^39^,^40^,^41^,^50^. Recombination afterglow contributes mainly to its power. However as indicated in Fig. 3, for the intensities over *I* ~ 6 × 10^20^ W/cm^2^ the behavior of X-ray power drastically changes, i.e. the power starts rising very non-linearly, approximately as ~*E*^4–5^ or ~*a*_0_^8–10^ (where *a*_0_ is the normalized laser field). This indicates that collision-less radiation, initiated by energetic electrons in Zone 1, becomes dominant. The power fitting is used in Fig. 3 to demonstrate only a very non-linear character of the power growth in our experiments. The fitting is not a universal; the power growth may be very sensitive to laser and target parameters; its analysis requires more measurements for different pulse and target conditions. Also, such a fitting is suitable only for collision-less radiation and is not suitable for the recombination afterglow. For lower energy laser pulses (*E* = 0.4 J in Fig. 3), the X-ray radiation emitted mostly during plasma recombination exceeds the collision-less portion, as expected. The afterglow X-ray radiation makes a kind of background which becomes small for *I* > 6 × 10^20^ W/cm^2^.

From Fig. 2(b) is also clear that for lower laser intensities *I* < 6 × 10^20^ W/cm^2^ the intensity of the X-ray spectra is very weak; the spectral structures are rather simple, and only intense spectral lines of neutral K_α_ and He_α_ lines with Li-like di-electronic satellites are prominent. Further increasing in the laser intensity results not only in an increase in the X-ray spectral intensity, but also in the appearance of new line features indicative of high X-ray flux interacting with high-density plasma. The appearance of intense “hollow-ion” emission lines between He_α_ and Ly_α_ clearly indicates that, at these laser intensities, there is significant X-ray pumping of the peripheral plasma from the central plasma resulting in photo-ionization of Al ions (see for details Refs. 17,32,33). This result indicates that the RDKR is reached at laser intensities around 10^21^ W/cm^2^.

Along with the spectral measurements, we carried out measurements of the energy distribution of energetic electrons along the direction of the incident laser beam^51^ with the use of a high magnetic field electron spectrometer as shown in Fig. 2(a). Typical electron spectra measured for the laser pulses intensity of ~10^21^ W/cm^2^ and Al foils with thickness of 2 and 6 µm are presented in Fig. 2(c,d). The results show a large quantity of electrons with 10 MeV energies, which are the source of powerful X-ray emission in the central plasma. By fitting the measured electron energy spectra in the 10–30 MeV range, where measurement errors are small, we determined the hot electron temperatures to be in the range of T_e,hot_ ~ 4–5.5 MeV and observed an increase of the hot electron temperature with the Al target thickness.

### Analysis of experimental results

The heating of bulk plasma by ultra-intense laser beams has been considered in Refs. 38, 39, 40, 41, where it was shown that the bulk of central part can be heated up to temperatures of 300–500 eV at the laser pulse intensities of our experiment, but at a higher laser pulse duration. However, the models presented there allow for radiation to grow only linearly with the pulse energy and thus cannot explain the non-linear growth of X-ray energy observed in our experiments. Indeed, dynamics of transverse heat transfer in targets irradiated by femtosecond powerful laser pulses has yet to be well understood. According to Refs. 38, 39, 40, 41 energetic electrons from the central plasma Zone heat the periphery plasma with axial temperature gradient <1 μm scale length^38^,^40^. Moreover, with the laser power growth the light pressure increases and makes the central Zone move away from the solid periphery decreasing efficiency of heating the periphery by energetic electrons. To our understanding, electron heating only may be not enough to initiate a non-linear growth of X-ray emission from plasma periphery and appearance of hollow ions there. Furthermore, with the laser pulse power growth the energies of energetic electrons increase reducing their direct energy deposition and saturating their current in the periphery; with the temperature increases the conductivity of plasma periphery decreases reducing the Ohmic heating by the return current^50^,^52^. Indeed as it was demonstrated in^17^,^33^ that only for photo-induced ionization the probability of KK hollow ion production is higher that the probability of KL hollow ion production.

**Figure 1:**
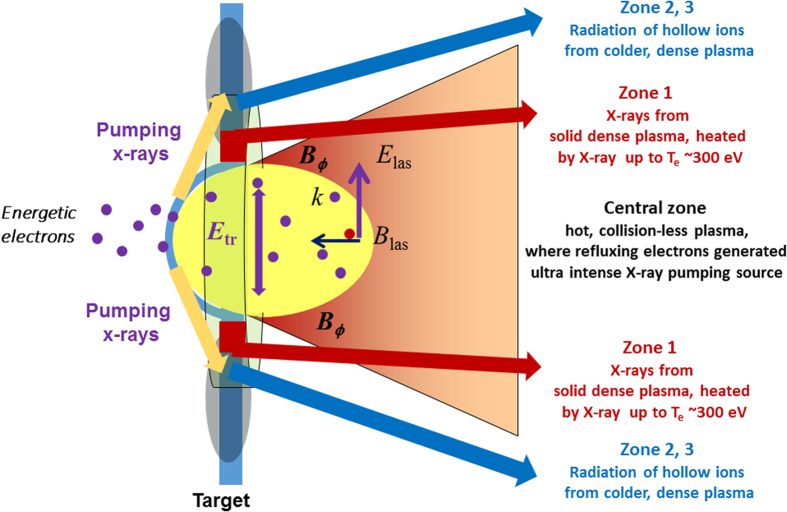
Scheme of generation of ultra-intense X-ray source in relativistic laser-produced plasma. Relativistic laser beam focuses on the foil (central Zone) and generates very intense bunches of fast electrons, which, due to strong refluxing in the expanded plasma, produce ultra-bright X-rays with a duration comparable to the laser pulse duration or slightly longer. Such transient RDKR X-ray source heats periphery of plasma up to hundreds eV temperature keeping it solid (Zone 1). At a greater distance from the center of the plasma (Zones 2 and 3) electron temperature drops to a few eV, and due to efficient pumping by X-ray source hollow ions are generated there. Plasma radiated by Zones 1–3 are measured by X-ray spectrometer.

We thus develop an alternative plasma model (See Fig. 1), initially proposed in Ref. 17, with a central Zone where the main relativistic laser pulse produces very intense bunches of fast electrons, which, due to strong refluxing in expanding plasma, generate ultra-bright X-rays with a duration comparable to the laser pulse duration or slightly longer, and with two parametric Macdonald function distributions of the radiation energy. Over the ~100 fs laser pulse duration, as energy is transferred from the laser fields to hot electrons, then to X-rays, and finally to thermal electrons, this transient RDKR goes from an initial cool, solid state with a radiation flux equivalent to a Planckian distribution with *T*_r_ > 1 keV to a warm (~50 eV), near-solid plasma with T_r_ ~ 1.5 keV, and ends as a hot thermal plasma with T_e_ ~ 300 eV, which radiates for a significantly longer duration (~10 ps) as it expands and radiatively cooled down.

Therefore, we suggest that an essential source of plasma heating in periphery Zone 1 is X-rays generated by refluxing electrons (see Fig. 1); the power of such X-rays increases non-linearly with the laser pulse energy. The heating temperature can be estimated with the use of photo-ionization cross-section within the Kramers approximation: 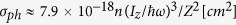, where *I*_z_ is the ionization potential, *ħω* is the energy of a photon, *n* is the quantum number, and *Z* is the ion charge. Assuming that plasma in this Zone is fully ionized by EUV radiation and with *n* = 2 and *Z* = 10, one can estimate the free path for the radiation with 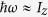 in solid density plasma: 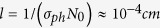. Thus, the absorption of X-rays is very strong and runs as a diffusion process from the center of the laser spot to the periphery. After photoionization, photo-ionized electrons have energy 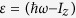, typically a few hundred eV. The thermal electron energy distribution, and therefore the electron temperature, strongly depends on the ‘pumping’ X-ray spectrum. Predicting this temperature requires a detailed simulation of the heat transfer process during the laser plasma interaction, which is beyond the scope of this paper. However, a qualitative estimation shows that the electron temperature in such plasma could be of the order of a few hundred eV. Such effective heating by X-ray radiation was also confirmed by simulations in Refs. 53, 54, 55 done for the XFEL irradiation of thin foils. Additionally, observed KL/KK hollow ions satellite ratios in the peripheral regions show that in our case X-ray pumped features are dominant over electron-pumped ones.

### Modeling of X-ray spectra

To understand better the physics of the radiation processes of relativistic plasma irradiated by ultra-short laser pulses with intensities ~10^21^ W/cm^2^, we have analysed the emission spectra using two collisional-radiative codes: the ATOMIC code^17^,^32^,^33^,^46^ and the hybrid structure Spectroscopic Collisional-Radiative Atomic Model SCRAM^32^,^48^. The modelling results for the experimental X-ray spectrum with 6.8 J of laser energy and a 6 μm target are presented in Figs. 4 and 5. The experimental X-ray spectrum obtained at the highest laser intensity ~10^21^ W/cm^2^ is in a very reasonable agreement with modelled spectra composed of three regions, or Zones, whose conditions and durations are consistent with the RDKR process described above: a cool, dense plasma heated by an intense local X-ray field that emits strongly over the laser duration followed by a hot, thermal, slightly expanded plasma that emits over a much longer duration. We also mention that such approaches have allowed successful modelling not only of the above mentioned spectrum, but also, for example, X-ray spectra for the case of 0.8 μm Al target irradiated by different laser energies of 4.4, 5.9 and 6.8 J (see Fig. 6).

We stress that the measured X-ray spectra are temporally and spatially integrated. Theoretical consideration^17^ of the hot electrons refluxing in the foil target suggest that the non-linear driving X-ray radiation is produced most strongly along the target surface (see Fig. 1) and thus we do not include it in our modelling of the X-rays measured at the spectrometer, which observes the target at ~45° to the surface.

Due to multiple reflection orders of the mica crystal, our X-ray spectrometer measures radiation from both 1450–1850 eV and 2210–2730 eV energy ranges. The emission of He_α_ and Ly_α_ and their di-electronic satellites in the 1450–1850 eV range comes from the “Zone 1” late-time plasma with T_e_ ~ 320 eV, which also contributes intense photo-recombination continuum emission in the 2210–2730 eV energy range. The early-time emission from the cool and dense plasma of Zones 2 and 3 pumped by the X-ray source has a lower intensity and contributes only around 10–15% of the total measured spectral intensity.

While modelling emission from the few-electron thermal K-shell ions in Zone 1 is fairly straightforward and there is excellent agreement between SCRAM and ATOMIC calculations, the emission from the colder radiation-driven Zones is more sensitive to model variations. Nonetheless, both models exhibit similar functional dependencies on the bulk electron temperature, self-opacity, and the distribution and intensity of the driving radiation field. In general, the charge state distribution of the Al ions is highly sensitive to the bulk electron temperature. Self-opacity, included only in the Zone 1 calculations of ATOMIC but in all Zones of the SCRAM calculations, will tend to reduce the KL/KK emission ratio. The intensity of the driving radiation impacts the absolute intensity of the emitted X-rays, the KL/KK ratio, and, to some extent, the charge state distribution. The spectral distribution of the driving radiation is a weaker lever on the X-ray emission but can, in principal, be varied resembling the physically motivated Macdonald radiation distribution representative of Compton and cyclotron spectra at similar intensities (see next Section) by including a brightness factor, which may be greater than unity, on a Planckian radiation field.

Based on this modelling, we have tabulated the intensity of secondary X-rays scattered from periphery of 0.8 and in 6 μm Al foil targets irradiated by laser pulses with different intensities (see Table 1). Apparently, the secondary X-rays have much lower power than the pumping X-rays from the plasma center. Table 1 shows that the intensity of the emission spectra scales less strongly with laser energy in the energy range of 1450–1850 eV (maximum growth is *E*^4.3^) than for the energy range 2210–2730 eV (maximum growth is *E*^6.1^). Such a difference may be explained by the fact that ~1.5 keV radiation is more strongly absorbed inside the volume of the solid dense plasma, or periphery. Since the free path of X-rays depends on their wavelength as ~*λ*^−3^, the absorption for radiation in 2.2–2.8 keV energy range is smaller, by a factor of 3. In cold Al, the attenuation length of 1.6 keV photons is about 1 micron and about 3 microns for 2.5 keV photons. Additionally, with increasing laser intensity, the number and the energy of fast electrons also increased, giving rise to more energetic X-ray photons.

As mentioned above, we found that the electron temperature of the dense late-time plasma in Zone 1 is heated up to ~300 eV by an ultra-intense X-ray source produced in central part of laser focusing Zone. We can roughly estimate what intensity of X-ray pumping source is needed to heat solid density plasma up to *T*_e_ ~ 300 eV using the energy conservation law: *I*_X-ray_ (W/cm^2^) > *N*_0_*V*[*E*_ion_ + <*Z*>*T*_e_]/(*Sτ*)*K*_abs_, where *N*_0_—atomic density, *V*—volume of heated plasma, *E*_ion_—energy needed for ionization of atom, <*Z*>—average ion charge, *S*-area through which plasma is heated, *τ*—X-ray heating time, *K*_abs_—part of the X-ray intensity absorbed by pumped foil. If we take into account Al foil thickness of 6 μm, X-ray pulse duration τ ~ 100 fs, average ions charge <*Z*> = 6 we obtain an X-ray intensity of pumping source as high as I_X-ray_ > 1.4 × 10^17^ (W/cm^2^). Such an estimated value of X-ray source intensity is in a good agreement with our observations of KK Hollow ions at the laser intensities approaching to 10^21^ W/cm^2^. Indeed, according with^15^,^17^ for observation of KK hollow ions spectra of Al it is required that X-ray source should have intensity at least 10^17^ W/cm^2^.

### PIC simulation of ultra-intense laser interaction with foils

The spectral characteristics of plasma radiation are of utmost interest for various applications. Unfortunately, there is not yet a complete theory of radiation from relativistic plasmas produced by a powerful laser pulse. In the classical approach^56^ the spectral and angular distributions of radiated energy given by the following expression


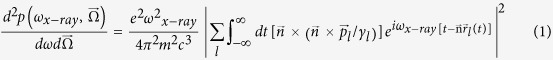


where *ω*_x–ray_ is X-ray frequency, *r*_l_(*t*), *p*_l_(*t*), and *γ*_l_(*t*) are radius, momentum, and relativistic factor of *l*-th plasma electron, are too complicated to be calculated in the frame of known simulation methods (for example, the particle-in-cell method^57^). Neglecting possible coherent emission in certain frequencies, we consider a typical spectrum of a single electron radiating with the characteristic frequency depending on the process. However for all processes, the frequency has the following form


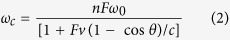


where *ω*_0_ is a frequency of a particular process: cyclotron, plasma or laser pulse frequency, *θ* is the angle of observation, *n* the number of harmonics, *v* the electron velocity, *F* is a Doppler parameter; for example, for Compton scattering the Doppler parameter *F* = *γ*^2^(1 + *v*/*c*)^2^/(1 + *a*_0_^2^/2).

It is obvious that the radiation spectrum depends on the angle of observation, *θ*. Since all radiation processes result in almost the same spectral shape^58^,^59^, one can approximate a spectrum using the frequency from Eq. (2) as


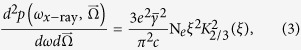


where *ξ* = *ω*_x–ray_/*ω*_c_, *K*_2/3_(*ξ*) is a Macdonald function, N_e_ is the number of energetic electrons in plasma. The approximation (3), as a two parametric function, can serve as a good approximation for detailed atomic kinetics simulations necessary to characterize spectroscopy data; *ω*_c_ and *N*_e_ are set as parameters; for *θ* = 0 this equation is exact. Equation (3) can be used for analysis of the RDKR in a two parameter parametric form.

To understand the role of radiation damping in the conditions of our experiments we performed 2D particle-in-cell simulation including collisions^60^ and radiation friction in its classical form^34^. Since as discussed in the introduction the radiation of shaped plasma can be stronger than that from a plane target, we performed two kind of simulation: (i) with a plain foil target and (ii) with a cranked foil target. Figure 7 shows results of calculations that were performed for a laser intensity *I* = 10^22^ W/cm^2^ and pulse duration 20 fs. In both cases the plasma is still far from the radiation damping regime, where radiation results in particle motion and an essential portion of laser energy is converted to X-rays in the central plasma^34^. In the present calculations only ~1% of laser energy is directly converted to X-rays. This means that the radiation dominant kinetic regime in the periphery plasma occurs for far lower laser intensities than the radiation damping regime.

## Discussion

We have measured a nonlinear increase in the X-ray intensity produced with increasing sub PWL intensities and shown that the spectrum of X-ray emission is consistent with a dense plasma that is heated by bright X-rays generated over the laser pulse duration from its initial cold state to final thermal temperatures of ~300 eV. Upon comparing the modelled and experimental spectra, we can conclude that for ultra-short femtosecond laser pulses RDKR begins at laser intensities ~10^21^ W/cm^2^ and is characterized by (i) heating of a solid density periphery plasma by X-ray absorption up to few-hundred-eV temperatures, and (ii) X-ray pumping of atomic and ion transitions, resulting in the appearance of hollow atom lines. Finally, the spectral fitting indicates that the plasma remains around solid density. These conclusions are consistent with comparisons of modelled and experimental spectra from thinner 0.8 μm Al foil with lower opacities. We believe that such a result could be explained only if we consider that refluxing electrons generate extremely strong soft X-ray radiation in the full thickness of used targets and that such radiation photo-ionizes the Al target to several hundred eV.

The results of the first direct measurements and analysis of X-ray power behavior in relativistic plasma irradiated by femtosecond laser pulses indicate that the Radiation Dominant Kinetics Regime is sharply achieved at pulse intensities around 10^21^ W/cm^2^, which are much lower laser intensities than those when the radiation damping starts play an essential role in the particle dynamics. The central part of laser plasma rapidly becomes an efficient X-ray source. This source heats solid density plasma periphery, with which re-radiation begins a non-linear growth as ~ *E*^4–5^. At laser intensities of *I* ~ 10^21^ W/cm^2^ generated X-ray intensity in the energy range of some keV exceeds 10^17^ W/cm^2^. Photoionization of ions and atoms by such an X-ray source becomes the dominant process in the kinetics of peripheral plasma, similar to the kinetics occurring under irradiation by XFELs. Our results strongly support theoretical predictions^34^,^35^,^36^,^37^,^38^ that with increasing laser intensities up to 10^22^–10^24^ W/cm^2^ a conversion efficiency of visible light both to soft and to hard X-rays may achieve ~ 30–50%, which opens a path towards many applications of recently developing petawatt-class lasers, particular with a high repetition rate and femtosecond pulse duration. Still more experiments in the broader range of laser energies and laser intensities, as well as new simulations, which should include self-consistently the processes of soft X-ray generation at such relativistic intensities and influence of X-ray radiation on the plasma heating, are strongly needed for better understanding and optimization of X-ray and gamma radiation generation.

## Methods

### Laser

The experiments are performed using the J-KAREN^49^, an optical parametric chirped-pulse amplification (OPCPA) Ti: Sapphire hybrid laser system at Kansai Photon Science Institute of Japan Atomic Energy Agency. Laser pulses have the energy on the target up to ~7 J, duration of 35 fs (full width at half maximum, FWHM, with respect to intensity), wavelength of 0.8 μm and a typical contrast of 10^10^ (with respect to power; at several picoseconds before the pulse peak). The contrast is improved with a saturable absorber inserted between the high-energy CPA oscillator and stretcher. The ultrafast Pockels cell with the extinction ratio of 200 is applied at 500 ps before the main pulse to secure the contrast on the nanosecond time scale. Using F/2.1 off-axis parabolic mirror, p-polarized laser pulses are focused onto a target at an incidence angle of 84°, producing a spot with the FWHM diameter of ~4.5 μm (FWHM) and the peak laser intensity of 10^21^ Wcm^−2^. For achieving a higher stability of laser parameters we performed shots after at least 30 minutes of laser system cooling. We checked the shot to shot pulse duration fluctuation before the experimental campaign and found that with the measurement period of 1 hour the pulse length fluctuation was smaller than ±15%. As we did shots in single shot mode, usually the time between shots did not exceed 1 hour. Because of this reason we believe that the fluctuation in laser pulse duration was smaller than this value. Energy was measured using a standard calorimeter in each laser shot with accuracy of about 10%. As a laser focusing alignment was done very carefully before each laser shot we estimate the possible fluctuation of focal spot size not more than 15%.

### Target

An Al foils with thickness 0.8, 2, 3, 6 μm are placed at the laser focus position with a 5 μm accuracy along the laser propagation axis, well within the Rayleigh length of 20 μm. As a laser focusing alignment was done very carefully before each laser shot, we estimate the possible fluctuation of focal spot size not more than 15%. Unfortunately such procedure limited our shot numbers to up to 5–6 per day.

### X-ray and electron spectra measurements

**Figure 2:**
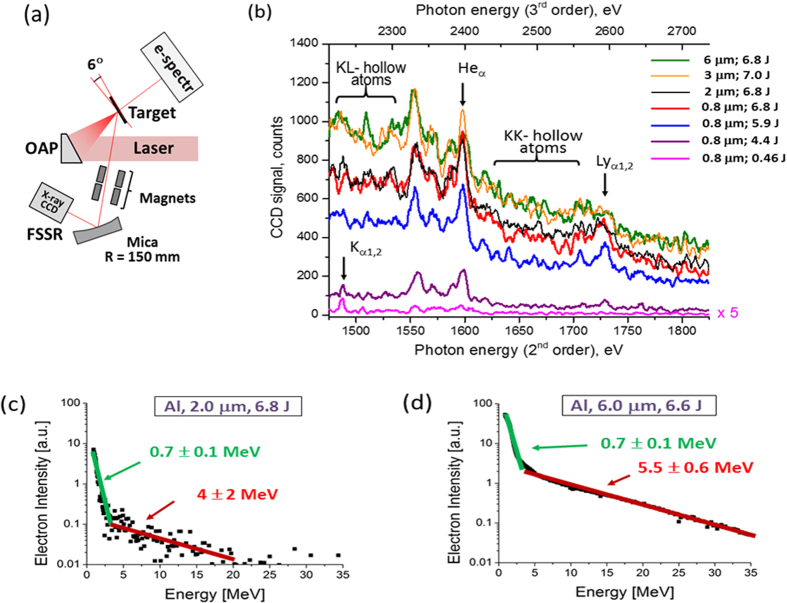
Scheme of observation and results. (**a**) Experimental set up. The laser beam is focused by an off-axis parabola almost perpendicular to the surface of foil and heats the resulting plasma. The produced plasma generates X-ray emission, which was measured by X-ray high luminosity spectrometer with high spectral resolution placed at 45° to the target surface. Electron spectra were measured by electron spectrometer placed from rear side of plasma perpendicular to the target surface; (**b**) Single shot, spatially- and temporally averaged Al ions K-shell spectra (raw data) emitted from foil targets with different thickness and laser energies (Intensity of spectra obtained by irradiation of laser pulse with energy 0.46 J is multiplied by factor 5); (**c**,**d**) Experimentally measured electron energy distribution in the case of irradiation of 2 and 6 μm thickness Al foils by maximum laser intensity of 10^21^ W/cm^2^. The increase of electron temperature of the hot electrons T_e,hot_ with the thickness of Al target is clearly observed.

**Figure 3:**
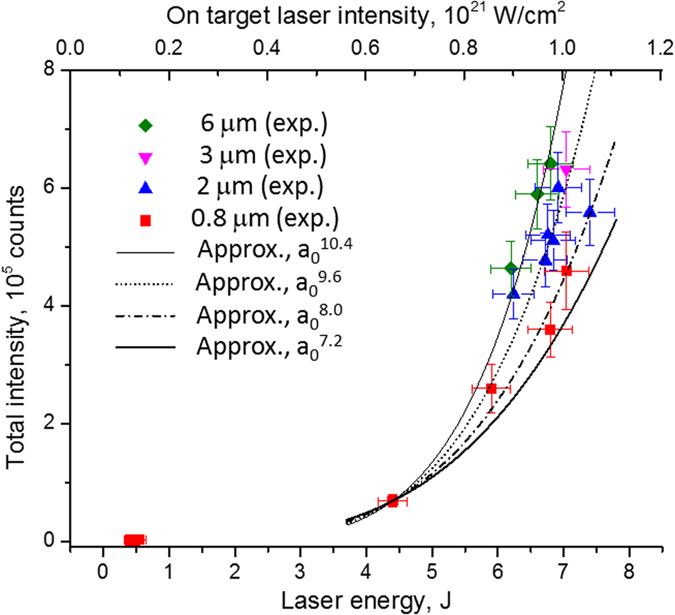
X-ray spectra intensities as a function of different parameters. Intensity of X-ray emission observed at X-ray CCD integrated over the energy range 1450–1850 eV and 2210–2730 eV (raw data) vs the laser intensity for Al foils with different thickness.

**Figure 4:**
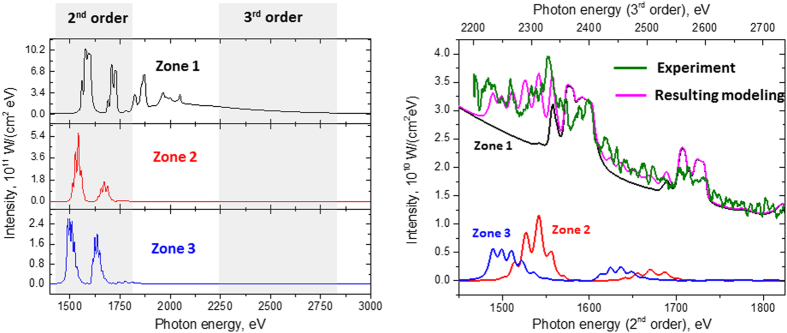
Theoretical spectra modelling by ATOMIC code. (**a**) Modelling was provided in assumption of plasma X-ray emission radiation from 3 plasma Zones (see Fig. 1). For all Zones 6 μm target thickness was used; Zone 1: *T*_e_ = 320 eV, *N*_e_ = 2 × 10^23^ cm^−3^; Zone 2: *T*_e_ = 55 eV, *N*_e_ = 3 × 10^23^ cm^−3^; Zone 3: *T*_e_ = 5 eV, *N*_e_ = 3 × 10^23^ cm^−3^. Intensity in Zone 2 was divided to factor 0.7 × 10^3^, intensity in Zone 3 was divided to factor 1.1 × 10^4^; Zones 2 and 3 do not include self-opacity effects but do include 1% hot electrons and a Planckian X-ray pump source with T_r_ = 1.5 keV, which is enhanced in Zone 3 by a factor of 10. The measured spectral regions registered by the second and third order reflections of the mica-crystal spectrometer are indicated in gray; (**b**) Comparison of theoretical and measured spectra, from 6.8 J laser pulse incident on 6 μm Al foil. The proportions of radiation input from the all 3 plasma Zones were adjusted to match the experimental spectrum, and suggest that the Zone 1 emission persists much longer than the emission from Zones 2 and 3.

**Figure 5:**
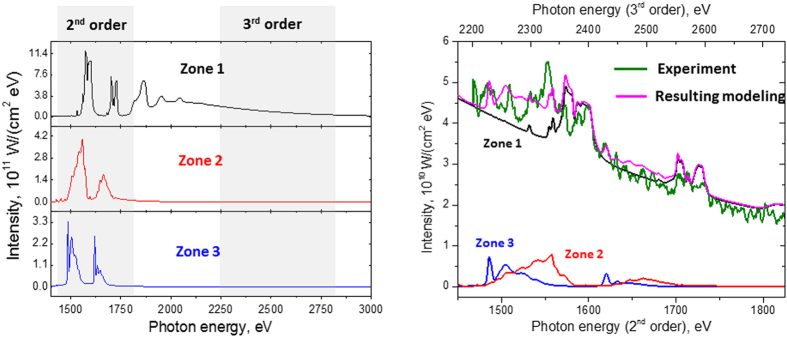
Theoretical spectra modelling by SCRAM code (a) Modelling was provided for plasma X-ray emission radiation from 3 plasma Zones (see Fig. 1). All Zones include self-consistent opacity effects for a 6 μm target thickness; Zone 1: *T*_e_ = 320 eV, *N*_e_ = 2 × 10^23^ cm^−3^; Zone 2: *T*_e_ = 50 eV, *N*_e_ = 3 × 10^23^ cm^−3^; Zone 3: *T*_e_ = 20 eV, *N*_e_ = 3 × 10^23^ cm^−3^. To fit the time-integrated spectrum, the intensity in Zone 2 was divided by 100 and the intensity in Zone 3 was divided by 660. Zones 2 and 3 include 1% hot electrons and a Planckian X-ray pump source with *T*_r_ = 1 and 1.5 keV, respectively. The measured spectral regions registered by the second and third order reflections of the mica-crystal spectrometer are indicated in gray; (**b**) Comparison of theoretical and measured spectra, from 6.8 J laser pulse incident on 6 μm Al foil. The proportions of radiation input from the 3 plasma Zones were adjusted to match the experimental spectrum, and suggest that the Zone 1 emission persists much longer than the emission from Zones 2 and 3.

**Figure 6:**
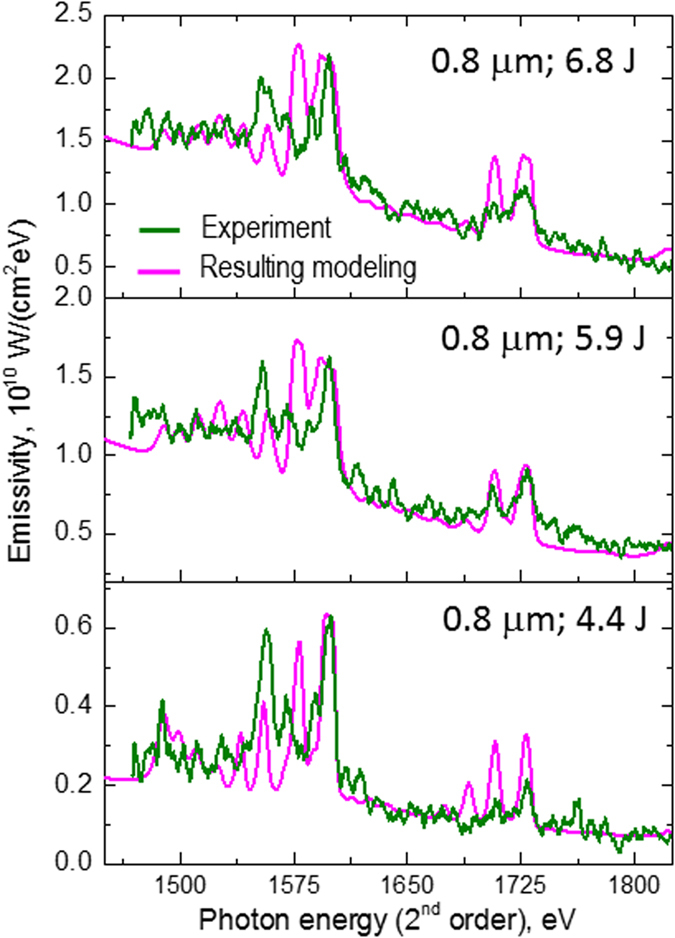
ATOMIC code modelling of spectra presented in Fig. 2 for the case of 0.8 μm targets irradiated by different laser energies. The modeling presented here has been performed using the same approach in each case (which has also been used for modelling experimental spectra in Fig. 4) - X-ray emission radiated from 3 plasma Zones: (**a**) Zone 1: *T*_e_ = 320 eV, *N*_e_ = 3 × 10^23^ cm^−3^; Zone 2: *T*_e_ = 55 eV, *N*_e_ = 3 × 10^23^ cm^−3^; Zone 3: *T*_e_ = 5 eV, *N*_e_ = 3 × 10^23^ cm^−3^, 1% hot electrons and a Planckian X-ray pump source with *T*_r_  = 1.5 keV, which is enhanced in Zone 3 by a factor of 10. (**b**) Zone 1: *T*_e_ = 300 eV, *N*_e_ = 3 × 10^23^ cm^−3^; Zone 2: *T*_e_ = 55 eV, *N*_e_ = 3 × 10^23^ cm^−3^; Zone 3: *T*_e_ = 5 eV, *N*_e_ = 3 × 10^23^ cm^−3^, 1% hot electrons and a Planckian X-ray pump source with *T*_r_  = 1.5 keV, which is enhanced in Zone 3 by a factor of 10. (**c**) Zone 1: *T*_e_ = 300 eV, *N*_e_ = 1 × 10^23^ cm^−3^; Zone 2: *T*_e_ = 80 eV, *N*_e_ = 2 × 10^23^ cm^−3^; Zone 3: *T*_e_ = 5 eV, *N*_e_ = 3 × 10^23^ cm^−3^, 1% hot electrons and a Planckian X-ray pump source with *T*_r_  1.2 keV, which is enhanced in Zone 3 by a factor of 10. The proportions of radiation input from the all 3 plasma Zones were adjusted to match the experimental spectrum, and suggest that the Zone 1 emission persists much longer than the emission from Zones 2 and 3.

**Figure 7:**
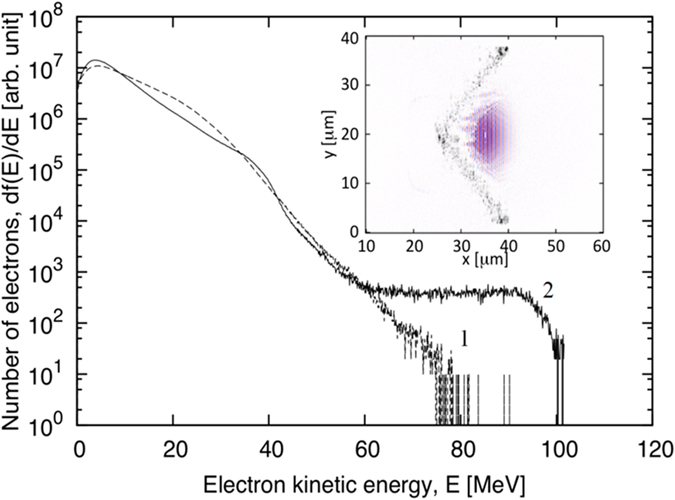
Two dimensional particle-in-cell simulation results. Electron energy distributions in 10 μm foil irradiated by a laser pulse with intensity *I* = 10^22^ W/cm^2^ and pulse duration *τ* = 20 fs. (1) a cranked foil as in the insert (2) a plane foil. Insert shows the ion distribution in the shaped foil and laser field during pulse irradiation.

For high-resolution spectroscopy measurements a Focusing Spectrometer with Spatial Resolution (FSSR)^61^ was implemented. The instrument was equipped by spherically bent mica crystal with a lattice spacing 2d ~ 19.94 Å and a radius of curvature of R = 150 mm. The crystal was aligned to operate at m = 2 of reflection to record K-shell emission spectra of multi-charged (He_α_ line of Al XII and Ly_α_ line of Al XIII) and neutral (i.e. K_α_ line) Al ions in 6.8–8.43 Å wavelength range, i.e., energy range from 1.45 to 1.85 keV. Simultaneously in 3rd order of mica crystal reflection recombination continuum in 2210–2730 eV energy range was registered. The FSSR spectral resolving power was approximately 3000. The spectrometer, observed the laser-irradiated front surface of the target at an angle of ~40° to the target surface normal and the target-to-crystal distance of 2045 mm. Background fogging and crystal fluorescence due to intense fast electrons were limited using 2 pairs of 0.5 T neodymium-iron-boron permanent magnets that formed a slit ~15 mm wide in front of the crystal (Fig. 2a). Spectra were recorded on X-ray Andor DX-420 back-illuminated CCD with 26 μm pixel size. The X-ray CCD was protected against exposure to visible light using two layers of 1 μm thick polypropylene coated from both sides with 0.2 μm Al. For comparison to the experimental data, forward processing of the calculated spectra was performed including filter absorption, CCD response, and a summation of the response of second and third reflection orders of mica crystals. Additionally, polypropylene filter of 6 μm thickness was used to reduce the noise level and reduce the signal from first order of mica crystal reflection. As we took special efforts for reproducible alignment of targets before each laser shot and careful cooling of laser amplifiers, the number of shots was strictly limited. Additionally we irradiated not only Al targets, but also targets with different thickness of Iron, Si, SAS^62^. In such cases the number of shots for each Al target thickness was not high and varied between 1 to 6 shots with different laser energies as it is presented in Fig. 3.

Measurements of the electron spectra were carried out with ESM which consists of a 0.4 T magnet, a phosphor screen, and a CCD camera as shown in Fig. 2(a). More details about the results of measurements of electron spectra at our laser intensities ~10^21^ W/cm^2^ may be found in Refs. 51,62,63.

### Atomic codes used for modeling

#### ATOMIC code

The ATOMIC calculations presented here used the same atomic model for Al as in previous modeling^17^,^32^. That is, large configuration sets were generated for all ions of Al, including configurations where most of the electrons are removed from the inner shells. The MUTA approach^46^ was used to calculate the large number of bound-bound transitions involving the levels associated with these configurations.

As previously discussed, it was found that it was not sufficient to assume that the X-ray radiation source was Planckian. Instead, use of either an “enhanced” Planckian source (where the amplitude of the distribution was increased by a factor of 10) or use of the Macdonald function as the radiation distribution function, produced emission spectra that were in better agreement with the measured spectra. Our ATOMIC calculations also included pressure ionization effects via the Stewart-Pyatt model^47^ as well as collisional broadening and included opacity effects on the strong lines via simple 1-D radiation transport models. Self-consistent opacity effects are included only in the Zone 1 calculations.

#### SCRAM code

The SCRAM calculations are based on a hybrid-structure model with extensive and convergent configuration sets. Ion Sphere continuum lowering that gradually destroys bound orbitals with increasing plasma density is implemented. The escape factor method is used to account for self-photo-pumping in all Zones, and the spectra are constructed using a mixture of fine-structure lines and unresolved transition arrays. Details are provided in previous work^32^. While the trends of modelled X-ray emission with variations in density, temperature, plasma size, and external radiation fields are quite similar to the ATOMIC calculations, the differing treatments of continuum lowering, opacity, and substructure of emission features lead to variations in the diagnosed conditions that should guide assessment of uncertainties.

#### Particle-in-cell (PIC) simulation code

Two dimensional particle-in-cell method including elastic collisions in the form of Langevin equation^58^,^59^ and radiation friction force in its explicit form given in^34^, code FPlaser2D, is used to calculate characteristics of a foil irradiated by a laser pulse with intensity *I* = 10^22^ W/cm^2^ and pulse duration 20 fs. We used a simulation box 40*λ* × 80*λ* with resolution *Δλ*/*λ* = 5 × 10^−3^ and with 100 particle per cell to simulate plasma with electron density *N*_e_ = 10^23^ cm^−3^ and thickness 10 μm. No ionization processes were included.

## Additional Information

**How to cite this article**: Faenov, A. Ya. *et al.* Nonlinear increase of X-ray intensities from thin foils irradiated with a 200 TW femtosecond laser. *Sci. Rep.* **5**, 13436; doi: 10.1038/srep13436 (2015).

## Figures and Tables

**Table 1:** Intensity of secondary X-rays in different energy ranges *vs* of laser pulse energy and target.

Laser and target parameters	Laser energy growth	Intensity (W/cm^2^) in the energy range 1450–1850 eV	Growth of X-ray intensity vs laser energy	Intensity (W/cm^2^) in the energy range 2210–2730 eV	Growth of X-ray intensity vs laser energy	Total intensity (W/cm^2^) in the investigated energy ranges	Growth of X-ray intensity vs laser energy
Ti:Sa laser, E = 6.8 J, I ~ 10^21^ W/cm^2^, 6 μm Al foil	1.55 × E	1.2.0 × 10^14^	E^4.2^ (a_0_^8.4^)	3.8 × 10^13^	E^6.1^ (a_0_^12.2^)	1.6 × 10^14^	E^4.6^ (a_0_^9.2^)
Ti:Sa laser, E = 6.8 J, I ~ 10^21^ W/cm^2^, 0.8 μm Al foil	1.55 × E	8.7 × 10^13^	E^3.5^ (a_0_^7.0^)	1.5 × 10^13^	E^4.0^ (a_0_^8.0^)	1.2 × 10^14^	E^3.5^ (a_0_^7.0^)
Ti:Sa laser, E = 5.9 J, I ~ 0.86 × 10^21^ W/cm^2^, 0.8 μm Al foil	1.34 × E	6.7 × 10^13^	E^4.3^ (a_0_^8.6^)	1.2 × 10^13^	E^5.3^ (a_0_^10.4^)	7.9 × 10^13^	E^4.4^ (a_0_^8.8^)
Ti:Sa laser, E = 4.4 J, I ~ 0.65 × 10^20^ W/cm^2^, 0.8 μm Al foil	E	1.9 × 10^13^	E	2.6 × 10^12^	E	2.2 × 10^13^	E
